# Draft Whole-Genome Sequence of the Purple Photosynthetic Bacterium Rhodopseudomonas palustris XCP

**DOI:** 10.1128/MRA.00855-18

**Published:** 2018-08-02

**Authors:** Amiera Rayyan, Terry Meyer, John Kyndt

**Affiliations:** a College of Science and Technology, Bellevue University, Bellevue, Nebraska, USA; b Department of Chemistry and Biochemistry, University of Arizona, Tucson, Arizona, USA; University of Delaware

## Abstract

Rhodopseudomonas palustris is known for its versatile metabolic capabilities and has been proposed for a wide range of innovative applications. Here, we report the genome sequence of strain XCP, as well as a whole-genome nucleotide comparison of R. palustris strains, which indicates the need for further differentiation of the known strains.

## ANNOUNCEMENT

Rhodopseudomonas palustris is a Gram-negative, purple, nonsulfur alphaproteobacterium that was initially identified by van Niel in 1944 (1). Since then, it has been isolated from a wide variety of environments, including aquatic sediments (2), sludge (3, 4), soils (5), alkaline waters (6), and eutrophic ponds (7), and it can be cultivated under several conditions. Therefore, R. palustris has potential in many applications ranging from hydrogen and electricity production (4, 8–10) to bioremediation of aromatic compounds, including the important environmental pollutant skatole, and the dehalogenation of carboxylic acids (11–13), and it has even been commercialized as a biostimulant for agriculture (14).

Several R. palustris strains have been sequenced since 2004 (13–15). The XCP strain was isolated in 1966 as a contaminant of a green bacterial culture in La Jolla, California, and labeled XCP for “ex Chlorobaculum parvum.” Its cytochrome C2 and C′ sequences were determined (16, 17), which clearly indicated that this strain was different from any of the other known R. palustris cultures and called into question the amount of diversity allowed within a species. In order to examine further potential differences in this strain, we isolated DNA from decades-old frozen cells using the GeneJET DNA purification kit (Thermo Scientific). The quantity and quality of DNA was determined using Qubit and NanoDrop and showed an absorbance at 260 nm (*A*_260_)/*A*_280_ ratio of 1.85. The DNA library was prepared using the Nextera DNA Flex library prep kit (Illumina). The genome was sequenced by an Illumina MiniSeq instrument using 500 μl of a 1.8 pM library. Paired-end (2 × 150-bp) sequencing generated 1,763,698 reads, yielding a total of 266.36 Mbp. The data were assembled *de novo* using the Velvet app (version 1.2.10) (18) within BaseSpace (Illumina). The assembled genome consisted of 66 contigs, with the largest contig being 1,081,439 bp, with an *N*_50_ of 134,556 bp. The GC content was 65.2%. The genome sequence was annotated using the Rapid Annotations using Subsystems Technology (RAST) server (version 2.0) (19), which found strain XCP to be 5,590,663 bp long and identified 42 contigs (with protein-encoding genes), 5,248 coding sequences (CDS), and 57 tRNAs.

There is a single operon of rRNA genes, with the small subunit showing 7 out of 1,468 differences from strains CG9 (15) and TIE-1 (20). Strain XCP has a complete set of Nap, Nir, Nor, and Nos genes for denitrification and the Sox genes for thiosulfate and sulfide utilization, as well as genes for nitrogen fixation, hydrogen uptake, carbon monoxide utilization, and Fe(II) oxidation. It also has cytochrome P450 genes for substrate hydroxylation and pyrroloquinoline quinone (PQQ)-dependent periplasmic dehydrogenases.

A JSpecies comparison (21) of the average percent nucleotide identity (ANLb) between XCP and other published R. palustris strains gave the following percentages: 88.1%, CG9; 88.0%, TIE; 88.0%, DX1; 81.8%, HA2; 81.3%, B29; 81.1%, B5; 78.2%, A53; 78.0%, JSC3B; and 77.9%, B18. These numbers are clearly below the proposed 95% cutoff for genome definition of a species, leaving only strains 2.1.6^T^ (not yet sequenced) and B5 within the confines of R. palustris. Furthermore, this suggests that XCP and most of the other nominal R. palustris strains studied to date should be recognized as separate species.

### Data availability.

This whole-genome sequencing project has been deposited at DDBJ/ENA/GenBank under the accession number QKQS00000000. The version described in this paper is version QKQS01000000.
